# Tuberculosis notifications among children and young adolescents, 2008–2019 compared to 2020–2023, Brazil

**DOI:** 10.2471/BLT.25.293446

**Published:** 2025-11-21

**Authors:** Antônio Candido Paiva Figueiredo dos Santos, Daniele Maria Pelissari, Marcos Otávio Brum-Antunes, Luiz Henrique Arroyo, Eduardo de Souza Alves, Renato T Stein, Leonardo Araújo Pinto, Fernanda Dockhorn, Costa, Frederico Friedrich, Marcelo Comerlato Scotta

**Affiliations:** a School of Medicine, Pontifícia Universidade Católica do Rio Grande do Sul (PUCRS), Avenida Ipiranga 6681, School of Medicine, 2nd floor, Porto Alegre, 90619-900, Rio Grande do Sul state, Brazil.; b National Tuberculosis Program, Health and Environment Surveillance Secretariat, Ministry of Health, Brasilia, Brazil.

## Abstract

**Objective:**

To evaluate the impact of the coronavirus disease 2019 (COVID-19) pandemic on tuberculosis-related notifications and mortality in Brazilian children and young adolescents aged 0–14 years.

**Methods:**

We conducted an ecological study using interrupted time series analysis with data from DATASUS, a nationwide open-access database of the Brazilian health ministry. We compared the notification rates of overall active tuberculosis, laboratory-confirmed tuberculosis, miliary and meningeal tuberculosis and tuberculosis-related deaths between the pre-pandemic (January 2008 to February 2020) and post-pandemic (January 2022 to December 2023) periods.

**Findings:**

Between 2008 and 2023, 43 216 tuberculosis notifications were recorded among children and young adolescents. The average annual notification rate per 100 000 population rose from 5.75 pre-pandemic to 8.37 post-pandemic, a 45.6% increase. Children younger than 1 year consistently had the highest rates. Laboratory-confirmed tuberculosis notifications totalled 12 557, with rates increasing from 1.55 per 100 000 pre-pandemic to 3.01 per 100 000 post-pandemic (94.2% increase). The average annual notification rate for miliary and meningeal tuberculosis increased from 0.22 to 0.29 per 100 000 (31.8%), and for tuberculosis-related deaths from 0.09 to 0.14 per 100 000 (55.6%). In 2023, laboratory-confirmed tuberculosis and miliary and meningeal forms had the highest rates (3.37 and 0.33 per 100 000, respectively), while deaths were the second highest on record (0.15 per 100 000).

**Conclusion:**

COVID-19 disruptions to tuberculosis services led to increased tuberculosis notification rates among Brazilian children and adolescents after the pandemic, due to higher transmission following a period of underdiagnosis. This setback hinders progress towards the End TB Strategy goals.

## Résumé

**Objectif:**

Évaluer l’impact de la pandémie de coronavirus 2019 (COVID-19) sur les notifications et la mortalité liées à la tuberculose chez les enfants et les jeunes adolescents brésiliens âgés de 0 à 14 ans.

**Méthodes:**

Nous avons mené une étude écologique à l’aide d’une analyse de séries chronologiques interrompues à partir des données de DATASUS, base de données en libre accès du ministère brésilien de la Santé. Nous avons comparé les taux de notification de la tuberculose active globale, de la tuberculose confirmée en laboratoire, de la tuberculose miliaire, de la méningite tuberculeuse et des décès liés à la tuberculose entre les périodes d’avant la pandémie (janvier 2008 à février 2020) et après la pandémie (janvier 2022 à décembre 2023).

**Résultats:**

Entre 2008 et 2023, 43 216 cas de tuberculose ont été signalés chez des enfants et de jeunes adolescents. Le taux annuel moyen de notification pour 100 000 habitants est passé de 5,75 avant la pandémie à 8,37 après la pandémie, soit une augmentation de 45,6%. Les enfants de moins d’un an présentaient systématiquement les taux les plus élevés. En tout, 12 557 cas de tuberculose confirmés en laboratoire ont été signalés, avec des taux passant de 1,55 pour 100 000 avant la pandémie à 3,01 pour 100 000 après la pandémie (soit une augmentation de 94,2%). Le taux annuel moyen de notification des cas de tuberculose miliaire et de méningite tuberculeuse est passé de 0,22 à 0,29 pour 100 000 habitants (soit une augmentation de 31,8%), et celui des décès liés à la tuberculose de 0,09 à 0,14 pour 100 000 habitants (augmentation de 55,6%). En 2023, la tuberculose confirmée en laboratoire, les formes miliaires et la méningite tuberculeuse ont enregistré les taux les plus élevés (respectivement 3,37 et 0,33 par 100 000 habitants), tandis que les décès ont atteint le deuxième niveau le plus élevé jamais enregistré (0,15 pour 100 000).

**Conclusion:**

Les perturbations causées par la COVID-19 dans les services de lutte contre la tuberculose ont entraîné une augmentation des taux de notification de la tuberculose chez les enfants et adolescents brésiliens après la pandémie, en raison d’une transmission plus élevée après une période de sous-diagnostic. Ce revers entrave les progrès vers la réalisation des objectifs de la Stratégie de l’OMS pour mettre fin à la tuberculose.

## Resumen

**Objetivo:**

Evaluar el impacto de la pandemia de la enfermedad por coronavirus de 2019 (COVID-19) en las notificaciones relacionadas con la tuberculosis y en la mortalidad de niños y adolescentes jóvenes brasileños de 0 a 14 años.

**Métodos:**

Se realizó un estudio ecológico mediante análisis de series temporales interrumpidas con datos de DATASUS, una base de datos nacional de acceso abierto del Ministerio de Salud de Brasil. Se compararon las tasas de notificación de tuberculosis activa en general, tuberculosis confirmada por laboratorio, tuberculosis miliar y meníngea, y muertes relacionadas con la tuberculosis entre los periodos prepandémico (enero de 2008 a febrero de 2020) y pospandémico (enero de 2022 a diciembre de 2023).

**Resultados:**

Entre 2008 y 2023, se registraron 43 216 notificaciones de tuberculosis en niños y adolescentes jóvenes. La tasa media anual de notificaciones por 100 000 habitantes aumentó de 5,75 en el periodo prepandémico a 8,37 en el pospandémico, un incremento del 45,6%. Los niños menores de 1 año presentaron de forma constante las tasas más elevadas. Las notificaciones de tuberculosis confirmada por laboratorio ascendieron a 12 557, con un aumento de las tasas de 1,55 por 100 000 en el periodo prepandémico a 3,01 por 100 000 en el pospandémico (incremento del 94,2%). La tasa media anual de tuberculosis miliar y meníngea aumentó de 0,22 a 0,29 por 100 000 (31,8%) y la de muertes relacionadas con la tuberculosis de 0,09 a 0,14 por 100 000 (55,6%). En 2023, la tuberculosis confirmada por laboratorio y las formas miliares y meníngeas presentaron las tasas más altas (3,37 y 0,33 por 100 000, respectivamente), mientras que las muertes registraron el segundo valor más alto documentado (0,15 por 100 000).

**Conclusión:**

Las alteraciones causadas por la COVID-19 en los servicios de tuberculosis dieron lugar a un aumento de las tasas de notificación de tuberculosis en niños y adolescentes brasileños después de la pandemia, debido a una mayor transmisión tras un periodo de infradiagnóstico. Este retroceso dificulta los avances hacia los objetivos de la Estrategia Fin de la TB.

## ملخص

**الغرض:**

تقييم أثر جائحة مرض فيروس كورونا 2019 (كوفيد 19) على حالات الإبلاغ عن حالات السل، ومعدلات الوفيات لدى الأطفال والمراهقين البرازيليين الذين تتراوح أعمارهم بين 0 و14 عامًا.

**الطريقة:**

قمنا بإجراء دراسة بيئية باستخدام تحليل السلاسل الزمنية المتقطعة، مع بيانات من قاعدة بيانات DATASUS، وهي قاعدة بيانات وطنية متاح الوصول إليها، وتابعة لوزارة الصحة البرازيلية. قمنا بمقارنة معدلات الإبلاغ عن حالات السل النشط بشكل عام، وحالات السل المؤكدة مختبريًا، والسل الدخني والسحائي، والوفيات المرتبطة بالسل بين فترتي ما قبل الجائحة (يناير/كانون ثاني 2008 إلى فبراير/شباط 2020)، وما بعدها (يناير/كانون ثاني 2022 إلى ديسمبر/كانون أول 2023).

**النتائج:**

بين عامي 2008 و2023، تم تسجيل 43216 بلاغًا بالسل بين الأطفال والمراهقين. وارتفع متوسط معدل الإبلاغ السنوي لكل مائة ألف نسمة من 5.75 قبل الجائحة إلى 8.37 بعدها، بزيادة قدرها %45.6. وسجّل الأطفال الذين تقل أعمارهم عن سنة واحدة أعلى المعدلات في كل الأوقات. وبلغ إجمالي حالات الإبلاغ عن السل المؤكدة مختبريًا 12557 إخطارًا، مع ارتفاع المعدلات من 1.55 لكل مائة ألف قبل الجائحة، إلى 3.01 لكل مائة ألف بعدها (زيادة قدرها %94.2). ارتفع متوسط معدل الإبلاغ السنوي عن حالات السل الدخني والسحائي من 0.22 إلى 0.29 لكل مائة ألف (%31.8)، وعن الوفيات المرتبطة بالسل من 0.09 إلى 0.14 لكل مائة ألف (%55.6). في عام 2023، سجلت حالات السل الدخني والسحائي المؤكدة مختبريًا أعلى المعدلات (3.37 و0.33 لكل مائة ألف على التوالي)، بينما سجلت الوفيات ثاني أعلى معدل مسجل (0.15 لكل مائة ألف).

**الاستنتاج:**

أدت الاضطرابات التي أحدثها كوفيد 19 في خدمات مكافحة السل إلى زيادة معدلات الإبلاغ عن حالات السل بين الأطفال والمراهقين البرازيليين بعد الجائحة، وذلك بسبب ارتفاع معدل انتقال العدوى بعد فترة من التشخيص الناقص. ويعوق هذا التراجع التقدم نحو تحقيق أهداف استراتيجية القضاء على السل.

## 摘要

**目的:**

旨在评估 2019 冠状病毒病 (COVID-19) 大流行疫情对巴西境内年龄介于 0-14 岁之间的儿童和青少年结核病相关通报案例数和死亡人数的影响。

**方法:**

通过搜索巴西卫生部推出的全国开放获取数据库 DATASUS，我们利用所得数据以间断时间序列分析法开展了生态学研究。我们对比了大流行疫情出现前（2008 年 1 月至 2020 年 2 月）和大流行疫情出现后（2022 年 1 月至 2023 年 12 月）这两个时间段内活动性结核病、实验室确诊结核病、粟粒性与脑膜结核病以及结核病相关死亡人数的总体通报率。

**结果:**

在 2008 年至 2023 年期间，记录的儿童和青少年结核病通报案例数为 43,216 例。年均通报率从大流行疫情出现前的十万分之 5.75 上升至大流行疫情出现后的十万分之 8.37，增幅为 45.6%。1 岁以内婴儿的通报率始终处于最高水平。实验室确诊结核病通报案例数总计为 12,557 例，通报率从大流行疫情出现前的十万分之 1.55 上升至大流行疫情出现后的十万分之 3.01（增幅为 94.2%）。粟粒性与脑膜结核病的年均通报率从十万分之 0.22 上升至十万分之 0.29（增幅为 31.8%），而结核病相关死亡人数的年均通报率从十万分之 0.09 上升至十万分之 0.14（增幅为 55.6%）。2023 年，记录显示，实验室确诊结核病和粟粒性与脑膜结核病的通报率（分别为十万分之 3.37 和 0.33）最高，而结核病相关死亡人数的通报率（十万分之 0.15）排名第二。

**结论:**

COVID-19 扰乱了结核病相关卫生服务系统，在漏诊现象持续一段时间后，结核病传播率有所升高，从而导致大流行疫情出现后的巴西儿童和青少年结核病通报率明显上升。这种倒退现象阻碍了“终止结核病战略”目标实现的步伐。

## Резюме

**Цель:**

Оценить влияние пандемии коронавирусной инфекции 2019 г. (COVID-19) на связанные с туберкулезом уведомления и смертность среди бразильских детей младшего и раннего подросткового возраста (от 0 до 14 лет).

**Методы:**

Авторы провели экологическое исследование с применением анализа прерванных временных рядов, данные для которого были взяты из общенациональной базы данных с открытым доступом DATASUS Министерства здравоохранения Бразилии. Сравнивалась частота уведомлений в целом о случаях активного, лабораторно подтвержденного, милиарного и менингеального туберкулеза, а также связанных с этим заболеванием смертях в период до начала пандемии (с января 2008 г. по февраль 2020 г.) и после нее (с января 2022 г. по декабрь 2023 г.).

**Результаты:**

В период с 2008 по 2023 г. всего было зарегистрировано 43 216 уведомлений о туберкулезе у детей младшего и раннего подросткового возраста. Средняя частота ежегодных уведомлений на 100 000 человек населения возросла с 5,75 перед началом пандемии до 8,37 после нее, то есть рост составил 45,6%. У детей младше 1 года во всех случаях наблюдались наивысшие показатели. Число уведомлений о лабораторно подтвержденных случаях туберкулеза составило в общей сложности 12 557, и их частота выросла с 1,55 на 100 000 человек населения перед пандемией до 3,01 на 100 000 человек после нее (рост составил 94,2%). Среднегодовой показатель уведомлений о случаях милиарного и менингеального туберкулеза вырос с 0,22 до 0,29 на 100 000 человек населения (31,8%), а показатель смертности в связи с этим заболеванием – с 0,09 до 0,14 на 100 000 человек (55,6%). По состоянию на 2023 г. наиболее высокие показатели частоты наблюдались для лабораторно подтвержденных случаев туберкулеза и для милиарной и менингеальной форм туберкулеза (соответственно 3,37 и 0,33 случая на 100 000 человек населения), а за ними второе место занимала смертность (0,15 сообщения на 100 000 человек).

**Вывод:**

Сбои в оказании услуг пациентам с туберкулезом в связи с пандемией COVID-19 привели после ее окончания к росту сообщений о заболевании туберкулезом среди детей младшего и раннего подросткового возраста в Бразилии. Этот рост объясняется более высокой частотой передачи инфекции, последовавшей за периодом недостаточной диагностики. Такой регресс препятствует достижению целей стратегии по ликвидации туберкулеза.

## Introduction

Tuberculosis is one of the leading causes of death from a single infectious agent and among the top 10 causes of death worldwide.^1^ In 2023, an estimated 1.25 million people died from tuberculosis, including 161 000 people living with human immunodeficiency virus (HIV).^1^ According to the *Global tuberculosis report 2024*, the global number of tuberculosis case notifications decreased during the coronavirus disease 2019 (COVID-19) pandemic compared with the years before the pandemic, 7.1 million in 2019 compared with 5.8 million in 2020.^1^ Data for children aged 0–14 years showed a similar drop: 521 001 notifications in 2019 compared with 398 923 in 2020.^2^

This reduction in notifications may have been partly due to mitigation measures implemented to contain the spread of severe acute respiratory syndrome coronavirus 2 (the etiological agent of COVID-19), such as social distancing, hand hygiene and mandatory mask use. These measures may have affected the transmission of *Mycobacterium tuberculosis* as well as other respiratory pathogens.^3^^,^^4^ However, evidence suggests that the reduction was primarily due to interruptions in the tuberculosis care cascade, reflecting underdiagnosis and/or undertreatment.^5^^,^^6^

Mathematical modelling and analytical techniques have been used to predict the impact of COVID-19-related disruptions on tuberculosis services.^7^^–^^10^ For example, one model estimated that these disruptions could lead to a 20.0% increase in tuberculosis-related deaths up to 2024.^9^ Another study, which assessed the impact of COVID-19-related disruptions to health services on tuberculosis burden in India, reported that a 3-month suspension of tuberculosis services could lead to an estimated 9.2% increase in the number of cases and an estimated 3.6% increase in deaths up to 2025.^10^

Following the sharp decrease in global tuberculosis notifications in 2020 and 2021, the number of notifications has increased and now exceeds pre-COVID levels. In 2023, an estimated 10.8 million people developed tuberculosis worldwide compared with 7.1 million in 2019.^1^ This trend is a setback in achieving the goals of *The end TB strategy *of the World Health Organization (WHO), which aims to reduce incidence by 80%, reduce tuberculosis-related deaths by 90% and eliminate the catastrophic costs for tuberculosis-affected households by 2030.^5^^,^^6^^,^^11^ Understanding how these global setbacks affect vulnerable populations is critical. Children, in particular, may serve as an early indicator of these impacts because of their higher risk of progression to severe forms of tuberculosis.^12^

However, data on tuberculosis incidence rates in children after the COVID-19 pandemic are sparse.^13^ We therefore aimed to evaluate the impact of the COVID-19 pandemic on tuberculosis-related notifications and mortality in Brazilian children and young adolescents aged 0–14 years by comparing the pre-pandemic and post-pandemic periods.

## Methods

We conducted an ecological study using data from January 2008 to December 2023, extracted from DATASUS, a nationwide open-access database maintained by the Brazilian health ministry.^14^ The database contains anonymized, individual-level data on all notifications of active tuberculosis and tuberculosis-related deaths. We extracted data for all children and young adolescents diagnosed with tuberculosis (notified cases), including information on overall active tuberculosis, laboratory-confirmed tuberculosis, miliary and meningeal tuberculosis, and tuberculosis-related deaths. We stratified the study population into four age groups: < 1 year, 1–4 years, 5–9 years and 10–14 years. 

For this study, we defined the pandemic period as March 2020 to December 2021, corresponding to the implementation and gradual relaxation of mitigation measures.^15^

### Data sources

In Brazil, health workers officially notify all people diagnosed with tuberculosis in public and private health-care facilities to the Brazilian Notifiable Diseases Information System.^16^ Notification must be done within 7 days after diagnosis. After notification, patients receive tuberculosis treatment free of charge, provided exclusively through the public health system. The information system primarily records notifications and data on notifiable diseases listed in the National Compulsory Notification List of Diseases and Hazards, established by the Consolidation Law No. 4 of 28 September 2017.^17^ The tuberculosis module in the system includes specific data on the date of diagnosis, form of tuberculosis, microbiological confirmation and other sociodemographic information.

Deaths caused by tuberculosis are officially recorded as tuberculosis-related deaths in the Brazilian Mortality Information System. This system is primarily populated with death certificates issued by physicians and processed by municipal health authorities, who are trained in International Statistical Classification of Diseases and Related Health Problems, 10th revision (ICD-10) coding standards. Reported data are transferred to a state-level database. After local review and coding, data are consolidated at the state level and subsequently unified nationally by the health ministry, ensuring comprehensive coverage. This system is recognized for its high quality and completeness,^18^^,^^19^ and it provides microdata on deaths by age, sex, cause and residence of the deceased. Causes of death are reviewed and coded by trained municipal health workers, according to ICD-10, including tuberculosis-related ICD codes A15, A16, A17, A18 and A19. In cases of HIV–tuberculosis co-infection, deaths are recorded as HIV-related, in accordance with ICD-10 guidelines.

We obtained the total population by age from the Brazilian Institute of Geography and Statistics census data.^20^

### Outcomes

The primary outcome of interest was the diagnosis of active tuberculosis. Secondary outcomes included laboratory-confirmed tuberculosis, miliary and meningeal tuberculosis and tuberculosis-related deaths.

Among children younger than 10 years, the diagnosis of pulmonary tuberculosis is usually presumptive based on the clinical judgement of the attending physician. To improve diagnostic accuracy, the health ministry recommends the use of a scoring system developed in Brazil, which contains multiple criteria, such as clinical manifestations, radiological findings, history of close contact with a person with tuberculosis in the previous two years, nutritional status and results of tuberculin tests.^21^ This score has an estimated sensitivity of 89% and a specificity of 86%.^22^ Although the diagnosis of extrapulmonary tuberculosis may also be presumptive, it is more often laboratory-confirmed using methods that can also be used to confirm pulmonary tuberculosis. These methods include: (i) microbiological confirmation, defined as at least one smear positive for acid-fast bacilli; (ii) rapid molecular testing (Xpert® MTB/RIF, Cepheid, Sunnyvale, United States of America, from 2014 to 2019 or Xpert® MTB/RIF Ultra from 2019 to 2023); and (iii) solid culture using Löwenstein-Jensen or Ogawa-Kudoh media, or liquid culture using the mycobacteria growth indicator tube (Becton Dickinson, Sparks, USA).^23^ We considered miliary and meningeal tuberculosis diagnoses according to the reported notification data.

### Data extraction

We extracted data on 28 October 2024, using TabWin, the DATASUS tabulation software. We imported, processed and analysed all data in R (R Foundation, Vienna, Austria) and stored them in a database container (DBC) file format. To ensure data quality, two researchers independently reviewed the data, and a third researcher was consulted in case of discrepancies.

### Statistical analysis

We calculated notification rates by dividing the total number of notified cases according to the date of diagnosis by the age-specific population and multiplying by 100 000. We interpolated monthly population estimates between the census years of 2010 and 2023 to adjust the denominator. To assess significant differences, we calculated notification rate ratios with 95% confidence intervals (CIs), between epidemiological periods with COVID-19 mitigation measures and those without.

We compared the pre-pandemic period (January 2008 to February 2020) with the post-pandemic period (January 2022 to December 2023), and considered the pandemic period (March 2020 to December 2021) as an intervention phase. We also compared the three periods for the following outcomes: all active tuberculosis diagnoses; laboratory-confirmed tuberculosis, miliary and meningeal tuberculosis diagnoses; and tuberculosis-related deaths. We included all children and young adolescents in the main analyses and conducted subgroup analyses by age group.

We used an interrupted time series analysis to assess the impact of COVID-19 on tuberculosis control. Notifications and mortality were compared across three periods: pre-pandemic, pandemic and post-pandemic, assuming that the average and trend of the time series remain constant throughout the study period.

The linear model was specified as follows:
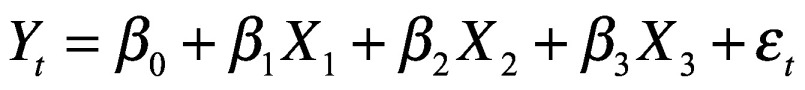
(1)where *Y_t_* is the outcome at time *t*; *X_1_* is a continuous variable representing the time in months during the pre-pandemic period; *X_2_* is a categorical variable indicating changes in the pandemic phase (0 is pre-pandemic, 1 is during the pandemic and 2 is post-pandemic); and *X_3_* is an interaction term between time and pandemic phase, representing the change in slope after the pandemic. *ε_t_* is the error term. The coefficients have the following interpretations: *β_0_* is the baseline level of the outcome at the start of the pre-pandemic period; *β_1_* is the monthly change in the outcome during the pre-pandemic period; *β_2_* is the change in the average level of the outcome during and after the pandemic compared to the pre-pandemic period; and *β_3_* is the change in the slope of the trend after the pandemic. Time was measured in months for all periods.

We performed all descriptive analyses and statistical tests in R, using the R packages readxl, dplyr, forecast, rstatix and read.dbc.

### Ethical considerations

The institutional review board exempted this study from approval because the DATASUS database is publicly available and contains no personal information.

## Results

**Fig. 1:**
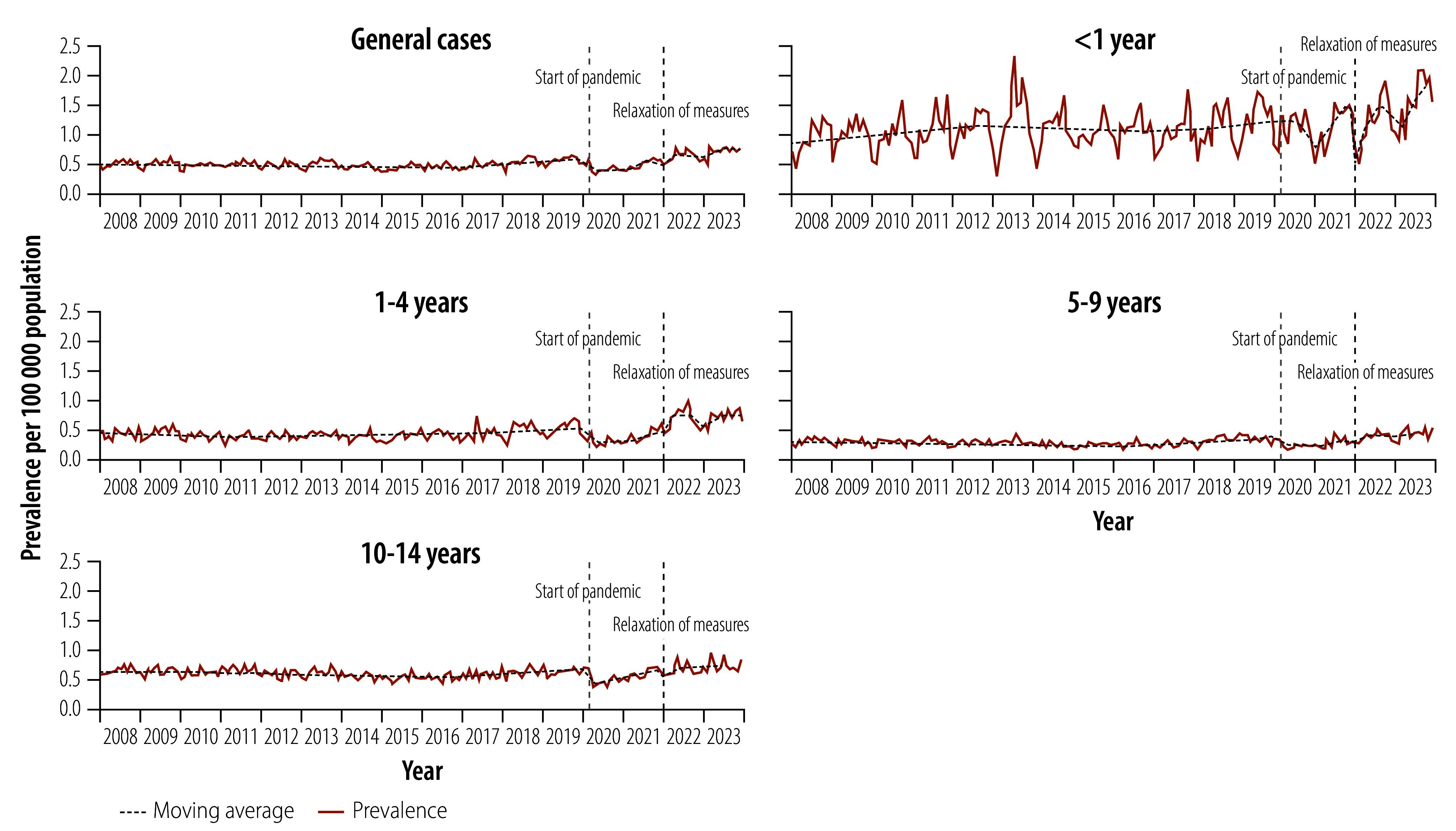
Notifications of tuberculosis cases among children aged 0–14 years, Brazil, 2008–2023

Note: we estimated the smoothed trends using locally weighted regression.

Between 2008 and 2023, 43 216 tuberculosis cases were notified among children and young adolescents. The average annual notification rate per 100 000 population increased from 5.75 in the pre-pandemic period to 8.37 in the post-pandemic period, representing a 45.6% increase. Across all years, children younger than 1 year had the highest notification rates of active tuberculosis. Up to 2021, children aged 10–14 years had the second highest rates; however, following the COVID-19 pandemic, rates among children aged 1–4 years surpassed those in the older age groups (Table 1). Tuberculosis notifications increased significantly between the pre-pandemic period and post-pandemic period (0.192 per 100 000 population; 95% CI: 0.142–0.235; Fig. 1). Annual notification rates for laboratory-confirmed tuberculosis, miliary and meningeal tuberculosis, and tuberculosis-related deaths also increased after the pandemic. In 2023, notifications of laboratory-confirmed tuberculosis and miliary and meningeal tuberculosis reached their highest recorded rates: 3.37 and 0.33 cases per 100 000 population, respectively. Tuberculosis-related deaths in 2023 were the second highest on record, at 0.15 deaths per 100 000 population; only in 2014 were more deaths reported (0.18 deaths per 100 000 population; Table 2). Detailed subgroup analyses by age and sex are available in the online repository.^24^

**Table 1:** Notifications of active tuberculosis in children and young adolescents, by age group, Brazil, 2008–2023

Year	No. of notified cases (cases per 100 000 population)				
	< 1 year	1–4 years	5–9 years	10–14 years	Total
2008	369 (11.03)	701 (5.28)	657 (3.83)	1287 (7.57)	3014 (5.94)
2009	402 (12.06)	728 (5.6)	608 (3.58)	1250 (7.46)	2988 (5.97)
2010	379 (11.38)	599 (4.68)	559 (3.34)	1129 (6.82)	2666 (5.39)
2011	417 (12.57)	568 (4.52)	590 (3.57)	1284 (7.87)	2859 (5.87)
2012	439 (13.27)	583 (4.74)	559 (3.43)	1095 (6.8)	2676 (5.57)
2013	488 (14.81)	614 (5.1)	567 (3.52)	1110 (6.99)	2779 (5.88)
2014	418 (12.67)	542 (4.59)	495 (3.11)	1014 (6.47)	2469 (5.29)
2015	392 (11.89)	539 (4.66)	457 (2.91)	936 (6.05)	2324 (5.05)
2016	427 (13.00)	562 (4.97)	476 (3.08)	916 (6.01)	2381 (5.26)
2017	393 (11.96)	587 (5.32)	497 (3.26)	986 (6.57)	2463 (5.53)
2018	431 (13.17)	652 (6.05)	585 (3.91)	1074 (7.27)	2742 (6.26)
2019	514 (15.79)	708 (6.73)	673 (4.58)	1094 (7.52)	2989 (6.95)
2020	428 (14.26)	390 (4.02)	459 (3.28)	820 (5.93)	2097 (5.2)
2021	430 (14.66)	501 (5.46)	485 (3.52)	970 (7.17)	2386 (6.09)
2022	460 (16.07)	803 (8.84)	651 (4.95)	1047 (7.87)	2961 (7.75)
2023	590 (21.52)	819 (9.23)	736 (5.88)	1187 (9.01)	3332 (8.99)

**Table 2:** Laboratory-confirmed tuberculosis, miliary and meningeal tuberculosis and tuberculosis-related deaths among children and young adolescents aged 0–14 years, Brazil, 2008–2023

Year	No. of notified cases (cases per 100 000 population)		
	Laboratory confirmed	Miliary and meningeal	Deaths
2008	629 (1.23)	125 (0.24)	50 (0.10)
2009	738 (1.47)	116 (0.23)	44 (0.08)
2010	661 (1.34)	100 (0.20)	49 (0.10)
2011	753 (1.54)	109 (0.22)	38 (0.07)
2012	638 (1.33)	97 (0.20)	44 (0.09)
2013	701 (1.48)	82 (0.17)	39 (0.08)
2014	687 (1.47)	97 (0.21)	86 (0.18)
2015	699 (1.52)	89 (0.19)	30 (0.06)
2016	714 (1.57)	98 (0.22)	31 (0.07)
2017	706 (1.58)	98 (0.22)	36 (0.08)
2018	863 (1.97)	134 (0.31)	35 (0.08)
2019	911 (2.11)	77 (0.18)	41 (0.09)
2020	718 (1.75)	87 (0.20)	41 (0.09)
2021	814 (2.04)	112 (0.24)	38 (0.09)
2022	1049 (2.67)	107 (0.26)	52 (0.12)
2023	1276 (3.37)	131 (0.33)	62 (0.15)

Note: notification data for active tuberculosis are presented in Table 1.

**Fig. 2:**
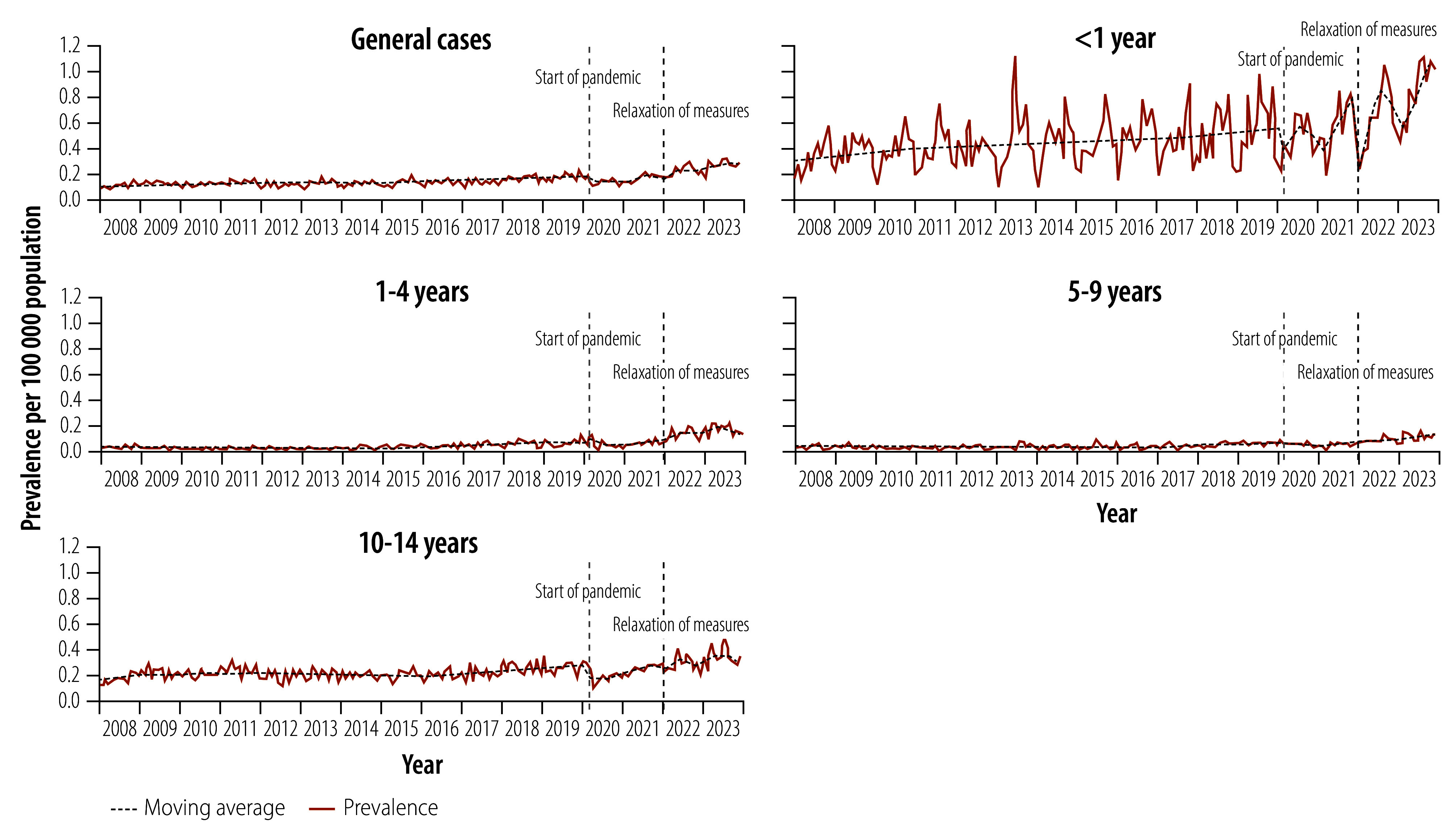
Notifications of laboratory-confirmed tuberculosis among children aged 0–14 years, Brazil, 2008–2023

Note: we estimated the smoothed trends using locally weighted regression.

During the study period, 12 557 laboratory-confirmed tuberculosis cases were notified among children and young adolescents. The average annual notification rate per 100 000 population increased from 1.55 in the pre-pandemic period to 3.01 in the post-pandemic period, representing a 94.2% increase. This upward trend was significant and consistent across age groups (0.078 per 100 000 population; 95% CI: 0.060–0.094; Fig. 2).

**Fig. 3:**
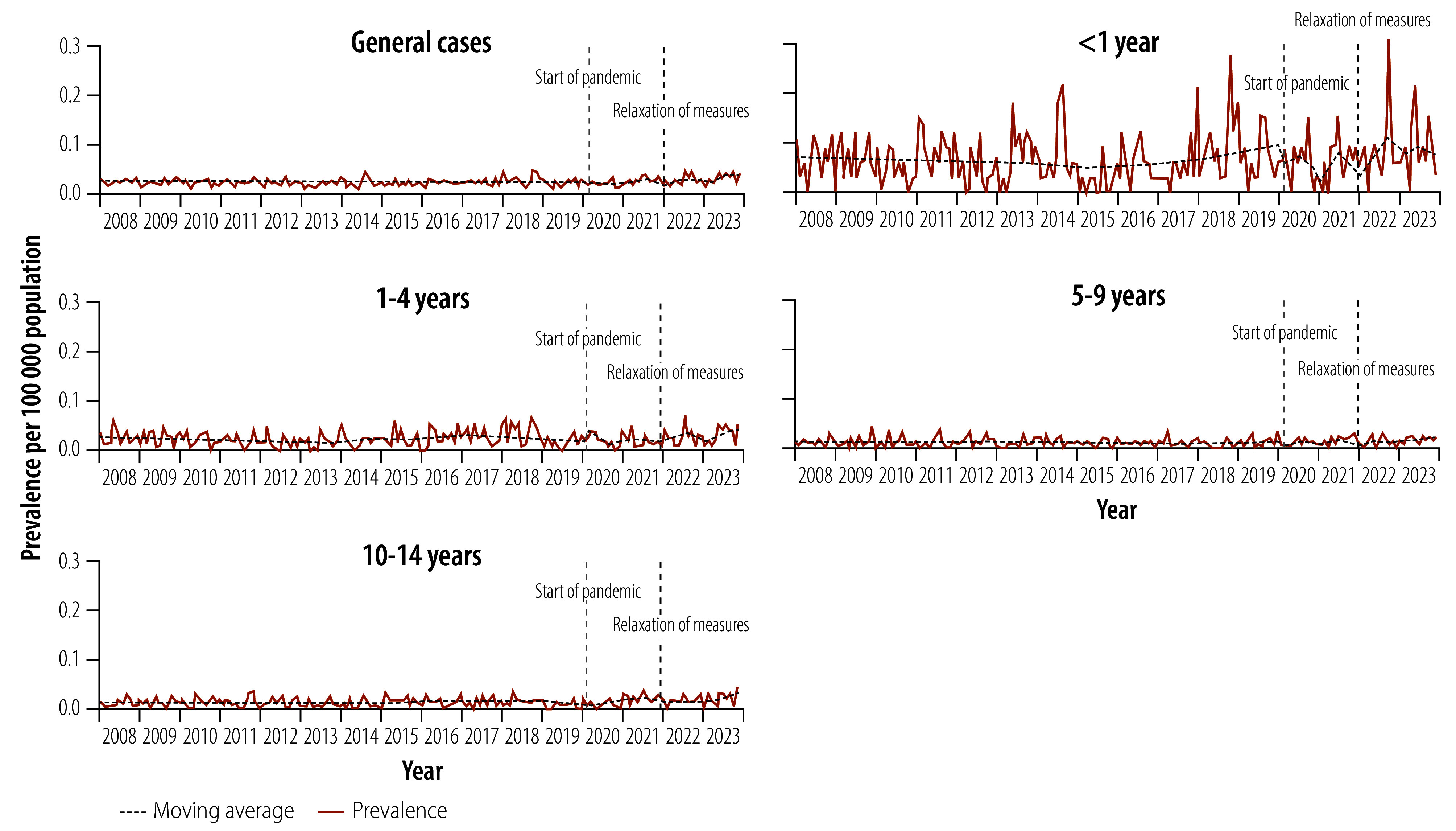
Notifications of miliary/meningeal tuberculosis among children aged 0–14 years, Brazil, 2008–2023

Note: we estimated the smoothed trends using locally weighted regression.

A total of 1659 miliary and meningeal tuberculosis cases were notified in children and young adolescents. The average annual notifications per 100 000 population increased from 0.22 in the pre-pandemic period to 0.29 in the post-pandemic period, representing a 31.8% increase. We observed no decline in reported cases during the pandemic (annual average of 0.23 cases per 100 000 population). The upward trend in notification rate was significant (0.003 per 100 000 population; 95% CI: 0.002–0.004; Fig. 3).

**Fig. 4:**
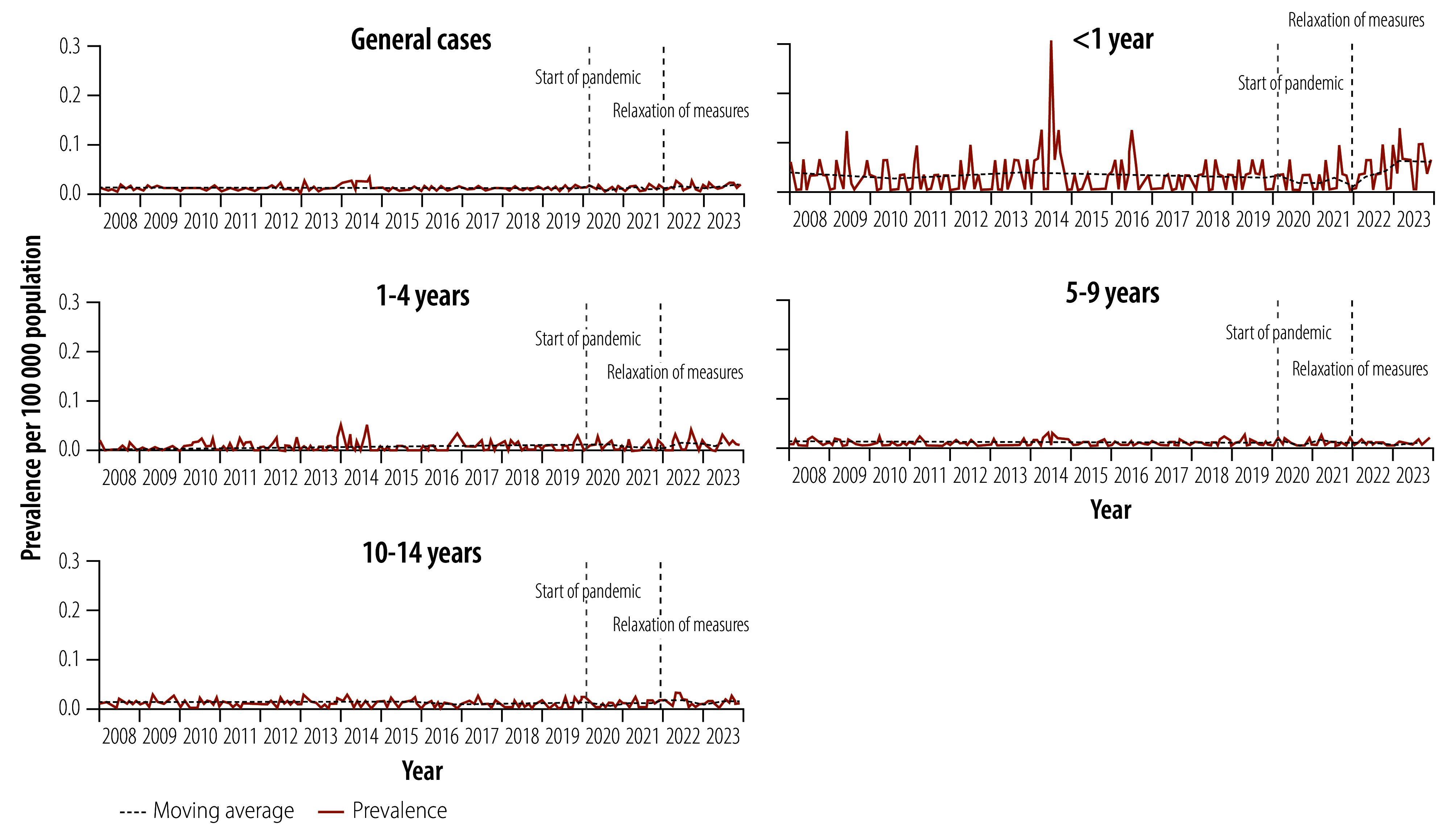
Notifications of tuberculosis-related deaths among children aged 0–14 years, Brazil, 2008–2023

Note: we estimated the smoothed trends using locally weighted regression.

During the study period, 716 children and young adolescents died from tuberculosis. The average annual notification rate of tuberculosis-related deaths per 100 000 population increased from 0.09 in the pre-pandemic period to 0.14 in the post-pandemic period, representing a 55.6% increase. As with the other outcomes, the upward trend in notification rate was also significant (0.003 per 100 000 population; 95% CI: 0.001–0.007; Fig. 4).

## Discussion

Here, we report decreased tuberculosis notifications among children and young adolescents in Brazil during the COVID-19 pandemic, followed by increased notification in the two years after the lifting of stringent mitigation measures. These findings are consistent with the 2024 epidemiological report on children and adolescents from the Brazilian health ministry.^25^ In the general population, data from other countries in the Region of the Americas also showed that the notifications in 2022–2023 were similar to or higher than the pre-pandemic levels.^1^ In contrast, notifications in the WHO European Region showed a downward trend during the same period. 

After the onset of the COVID-19 pandemic, estimated global tuberculosis-related deaths in the general population increased. The global number of estimated deaths did not fall below pre-pandemic levels until 2022 and 2023 (1.34 million deaths in 2019 versus 1.32 million in 2022 and 1.25 million in 2023).^1^ In the Region of the Americas, deaths in the general population only began to decline in 2023, although they remained above pre-pandemic levels.^1^ In contrast, our data show that deaths among children and young adolescents continued to rise through 2023, highlighting the need for more data on this population to ensure that policy responses consider all age groups. 

The reported impact of nonpharmacological measures implemented to mitigate COVID-19 on the transmission of other infectious agents has been heterogeneous.^26^^,^^27^ For example, the detection of respiratory syncytial virus decreased, while the incidence of rhinovirus increased.^26^^,^^27^ Evidence shows that the global incidence of tuberculosis declined during periods of stringent movement restrictions. This decrease was mainly attributed to disruptions across all stages of the tuberculosis care cascade, most likely reflecting underreporting rather than a true reduction in incidence.^1^^,^^11^^,^^28^^,^^29^ This hypothesis is reinforced by findings of low to no reduction in miliary and meningeal tuberculosis during the pandemic,^30^ which align with our data showing increased notifications of these forms after the easing of mitigation measures. These forms are typically severe and almost always require hospitalization, making them easier to detect and notify even during service disruptions. The stable incidence of these severe forms suggests that the observed decline in overall notifications was driven by underreporting of less severe cases rather than a true decrease in disease burden. These findings, along with increased tuberculosis-related deaths among children and young adolescents in 2022 and 2023, may be early markers of care disruptions during the COVID-19 pandemic.^31^ Such disruption would be expected to result in an early rise in all forms of tuberculosis among children,^32^ given their distinct progression and clinical presentation. One contributing factor may have been the decrease in the overall vaccination coverage in Brazil during the pandemic, including coverage of bacille Calmette–Guérin vaccine, which protects children from severe forms of tuberculosis.^33^

Our study adds to the existing literature on the impact of the COVID-19 pandemic on the epidemiology of tuberculosis in children. A global study using WHO-collated notification data compared observed childhood tuberculosis notifications in 2020 with predicted notifications based on trends from 2014–2019. All age groups experienced a reduction in notifications, with children aged 0–4 years showing the largest decrease (35.4%; 142 525 observed notifications versus 220 794 predicted notifications), followed by those aged 5–14 years (27.7%; 256 398 observed notifications versus 354 578 predicted notifications).^2^ Comparing the period 2010–2019 with 2020–2021, an Italian cohort study showed a decrease in tuberculosis notifications and diagnoses, and an increase in the incidence of severe forms according to Wiseman’s classification.^13^ In our study, the increase in notifications may be explained by Brazil being a high-tuberculosis-burden country, unlike Italy.

Resumption of tuberculosis services depends on several factors, such as the level of disruption of services due to the COVID-19 response, political commitment and availability of resources.^34^ The increase in notifications after the lifting of the mitigation measures in Brazil, as shown here and in the 2024 epidemiological report,^25^ indicates that implementation of tuberculosis control policies must be reinforced, considering the Brazilian heterogeneous territorial specificities. A possible explanation for the increase in overall tuberculosis notification rates could be the interruptions in the diagnostic and treatment cascade among adults,^35^^–^^37^ leading to increased transmission in households and the communities. Findings from a systematic review show that children exposed to a household member with tuberculosis were 3.79 times (95% CI: 3.01–4.78) more likely to be infected than unexposed children.^35^ These findings point to household transmission as a likely setting for the increase in notifications among children, suggesting the need to strengthen household-level interventions to decrease childhood tuberculosis and minimize the disruption in the care cascade for tuberculosis contacts.^38^

This study has some limitations. First, we used public secondary notification data, which may be subject to missing data or underreporting. Due to uncertainty regarding data fluctuations throughout the study period, we used the notification rate as the primary outcome of the study, which does not reflect the true incidence. However, most estimates on underreporting suggest that the underreporting in Brazil is usually low.^39^ Second, data on deaths extracted from the mortality database could also have issues, such as inadequate coverage and misclassifications. However, several quality assessments have found this database to be of high quality and usability.^18^^,^^19^ Third, the diagnosis of pulmonary tuberculosis in children is primarily presumptive. Nonetheless, notifications of laboratory-confirmed and miliary and meningeal tuberculosis showed similar trends. Fourth, the introduction of rapid molecular tests for tuberculosis during the study period might bias our findings. However, the increase in the incidence of all other forms reinforces the upward trend. Lastly, the DATASUS database has not been updated since 2023. Even if updated, it is usually based on a small number of remaining cases, which would not substantially change our data or alter the observed trends, but would make the differences more pronounced.

The rise in tuberculosis notifications among children and adolescents in Brazil after the easing of COVID-19 restrictions is a considerable setback to disease control and to achieving the goals of WHO’s* End TB strategy*.^40^ Improvements in integrated, patient-centred care policies are needed to restore the downward trend in childhood tuberculosis in Brazil and to reach the strategy’s milestones. As children are at increased risk of progression from latent to active tuberculosis, including miliary and meningeal forms, paediatric patients should be prioritized at all stages of the tuberculosis care cascade, especially regarding tuberculosis preventive treatment.
